# Distinct processes of lighting priors for lightness and 3-D shape perception

**DOI:** 10.1167/jov.21.6.1

**Published:** 2021-06-07

**Authors:** Yuki Kobayashi, Daniele Zavagno, Kazunori Morikawa

**Affiliations:** 1 School of Human Sciences, Osaka University, Suita, Japan; 2 Japan Society for the Promotion of Science, Tokyo, Japan; 3 Department of Psychology, University of Milano-Bicocca, Milan, Italy; 4 School of Human Sciences, Osaka University, Suita, Japan

**Keywords:** light-from-above prior, lightness/brightness, shape from shading, illusion, illumination

## Abstract

The visual system often relies on prior assumptions when interpreting ambiguous visual inputs. A well-known example is the light-from-above prior, which aids the judgment of an object's three-dimensional (3-D) shape (i.e., convex or concave). Recent studies have revealed that the light-from-above prior also helps solve lightness ambiguity. This study aimed to examine whether 3-D shape perception and lightness perception share the same lighting prior. The study participants performed two tasks: one focusing on lightness perception and another focusing on 3-D shape perception. The dominant directions of the assumed lighting were calculated from participants’ performance in the two tasks. The results showed that the assumed lighting direction for 3-D shape perception were considerably biased toward the left, whereas the one for lightness perception was almost from directly above. The clear difference between these two directions supports the hypothesis that the visual system uses distinct lighting priors for 3-D shape perception and lightness perception. Experiments 1 and 2 involved Japanese speaking participants and European participants, respectively. The Japanese language can be read and written both horizontally (i.e., left to right) and vertically (i.e., up to down) with lines progressing from right to left. Nevertheless, the two experiments still produced the same result, which suggests that the present finding is universal regardless of reading/writing direction.

## Introduction

**Figure 1.**
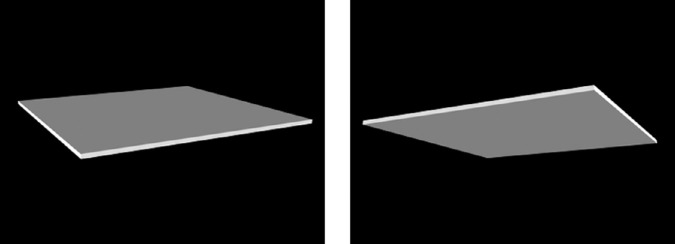
The two images are identical except for their rotation. The left panel appears slightly darker than the right, which can be attributed to their orientation and the lighting prior. These images were reproduced from Kobayashi and Morikawa (2019) with SAGE's permission.

When we see an object with an ambiguous three-dimensional (3-D) shape, we tend to address such ambiguity by assuming that illumination comes from above (Ramachandran, 1988; Sun & Perona, 1998, van Doorn, Koenderink, Todd, & Wagemans, 2012). Although the light-from-above prior assumption has been known to facilitate the perception of 3-D shapes, recent studies have also identified its relevance in lightness perception (Adams, Graf, & Ernst, 2004; Kobayashi & Morikawa, 2019; Menshikova, 2013). Kobayashi and Morikawa (2019) clearly demonstrated this effect by creating an illusion called the “inversion effect,” shown in Figure 1. The left and right panels of the figure are all identical except for their orientation, but the left panel surface appears slightly darker than the right panel. This illusion suggests that the visual system estimates and discounts more intense overhead illumination for the upward-facing surface on the left; hence, the slight darkening of the surface's lightness. The inversion effect of lightness was a novel demonstration of the role the light-from-above prior plays in lightness perception in the absence of any specific illumination cues.

We sought to empirically address the following question: if two different perceptual processes—3-D shape perception and lightness perception—involve a prior lighting assumption, do they also share the same prior, or do they use two independent ones?

Studies investigating the assumed lighting used in 3-D shape perception have found that its dominant direction is not directly from above, rather it is biased slightly toward the left (e.g. Mamassian & Goutcher, 2001; McManus, Buckman, & Woolley, 2004; Metzger, 2006; Sun & Perona, 1998). Some tasks using different stimuli have confirmed this left-biased tendency (Andrews, Aisenberg, d'Avossa, & Sapir, 2013; Gerardin, Montalembert, & Mamassian, 2007; Sun & Perona, 1998; Thomas, Nardini, & Mareschal, 2010), although its ecological origin is unclear. The fact that the lighting prior is not necessarily fixed directly overhead implies its flexibility and modifiability, and thus, one may hypothesize that its direction might vary depending on the visual tasks (e.g. judgments on shape or lightness).

Assumed lighting direction measurements by 3-D shape, perception studies have revealed significant differences between groups. For example, right- and left-handers prefer different lighting directions (Sun & Perona, 1998). Sun and Perona (1998) speculated that this phenomenon might be associated with one's experience in writing, during which an individual prefers illumination from the side opposite to their dominant hand so that their writing hand does not cast a shadow on the paper. However, other studies have failed to confirm the effect of handedness on the assumed illumination direction (Andrews, Aisenberg, d'Avossa, & Sapir, 2013; Mamassian & Goutcher, 2001; McManus et al., 2004). Andrews et al. (2013) found that the assumed lighting direction is affected by reading and writing experiences because Hebrew participants, who read and write from right to left, showed a much weaker (or lack of) leftward bias compared with English-reading participants, who showed a strong left bias. Developmental effects have been supported by several studies (e.g. Stone, 2011, Stone & Pascalis, 2010; Thomas, Nardini, & Mareschal, 2010). Adams's group demonstrated that even short-term training sessions in an experimental room can alter the assumed lighting direction (Adams et al., 2004; Adams, Kerrigan, & Graf, 2010; Kerrigan & Adams, 2013). The group used a type of training that coupled visual shape-from-shading stimuli with a haptic convexity/concavity feedback to ensure that the participants learned to associate particular light source directions with specific contexts. These studies highlight between-group differences in the lighting prior used to perceive 3-D shapes.

Adams (2007) took advantage of these differences to examine lighting priors. She attempted to clarify whether 3-D shape perception, visual search, and reflectance judgment use a common prior. Participants performed three different tasks, and the assumed lighting directions were calculated for each. If the participants used one common prior, the directions should be similar for all tasks. Indeed, the correlations between the tasks were significantly positive.

Adams (2007) results suggest that lightness and 3-D shape perception might share the same lighting prior. Other studies have also supported the shared-prior hypothesis. Knill and Kersten (1991) showed that perceived convexity changes lightness and illumination perception, indicating close relations among 3-D shape, lightness, and illumination. Other studies on visual material perception have also demonstrated the effect of 3-D shape perception on illumination impression and surface material perception, confirming the close associations between 3-D shape and lightness perception (Anderson & Kim, 2009; Marlow, Kim, & Anderson, 2017; Marlow, Mooney, & Anderson, 2019; Marlow, Todorović, & Anderson, 2015). Furthermore, Adams et al. (2004) found that a training session with information regarding stimuli shape and illumination can affect the lightness perception. This observation supports the hypothesis that the processes driving the shape and lightness perceptions use illumination information in the same manner. These studies indicate the plausibility of the shared-prior hypothesis.

However, studies have provided empirical evidence that the visual system may use different lighting priors to perceive lightness and 3-D shapes. Kerrigan and Adams (2013) showed that the visual system can learn and hold two different lighting priors. They had participants learn two lighting directions in two different contexts, after which they were able to modify their shape-from-shading estimates according to the two lighting directions. Adams (2008) showed that one's perception of gravity affects their assumed lighting direction in a shape judgment task but not in a visual search task despite both using similar stimuli. This suggested that the visual system could use different lighting priors even within the domain of shape-from-shading perception, providing further evidence for the independent-priors hypothesis.

The present study aimed to test the (in)dependence of the lighting priors used to perceive lightness and 3-D shapes. To understand the purpose of the experiments used in this study, let us consider Adams (2007) seminal work once more. Her results support the shared-prior hypothesis; however, the task in her experiment involved determining the cause of the lightness/brightness differences experienced in the stimuli (i.e., tetrahedrons that appeared illuminated by a distant light source). In other words, the participants were asked to evaluate whether the differences in the perceived intensity of the different faces of the tetrahedrons were caused by differences in illumination or pigmentation.

By contrast, the lightness task in the current study focused on how dark a surface appears and instead of asking why a surface is darker than another, the participants were asked to determine which of the two surfaces differently oriented in space is darker. Moreover, the stimuli we used, which were similar to the illusion depicted in Figure 1 (Kobayashi & Morikawa, 2019), did not include any cues to lighting direction other than the light-from-above prior.

In the present study, each participant performed a lightness perception task and a 3-D shape perception task, which were conducted with different types of stimuli. For each participant, the assumed lighting directions for the two tasks were calculated and examined to check for similarities or differences. If the two assumed lighting directions were the same and significantly positively correlated, such results would support the hypothesis of a single lighting prior for lightness and 3-D shape processing. By contrast, if the two directions were not correlated and showed different tendencies, such results would support the hypothesis of independent lighting priors for the two processes.

## Experiment 1

### Method

#### Participants

The present study included 26 naïve university students whose ages ranged from 20 to 28 years (*M* = 22.2, *SD* = 2.09, 9 women and 17 men). All participants spoke Japanese as their first language and had normal or corrected-to-normal visual acuity. One reported being left-handed. This study was approved by the Research Ethics Committee of the Osaka University School for Human Sciences and adhered to the Declaration of Helsinki.

#### Apparatus

A CRT monitor (Trinitron GDM-F520, SONY) with a 1600 × 1200 resolution was used. A ColorCAL II (Cambridge Research Systems) measured stimuli luminance. Viewing distance was fixed at 57 cm using a chin rest, and the experiment was run on PsychoPy2 (Peirce, 2007, 2009; Peirce et al., 2019). The participants performed the task in a dark room with no light source except for the monitor.

#### Stimuli and tasks

Each participant performed two tasks: a 3-D shape task and a lightness task. Their sequence was counterbalanced among the participants.

##### 3-D shape task

**Figure 2.**
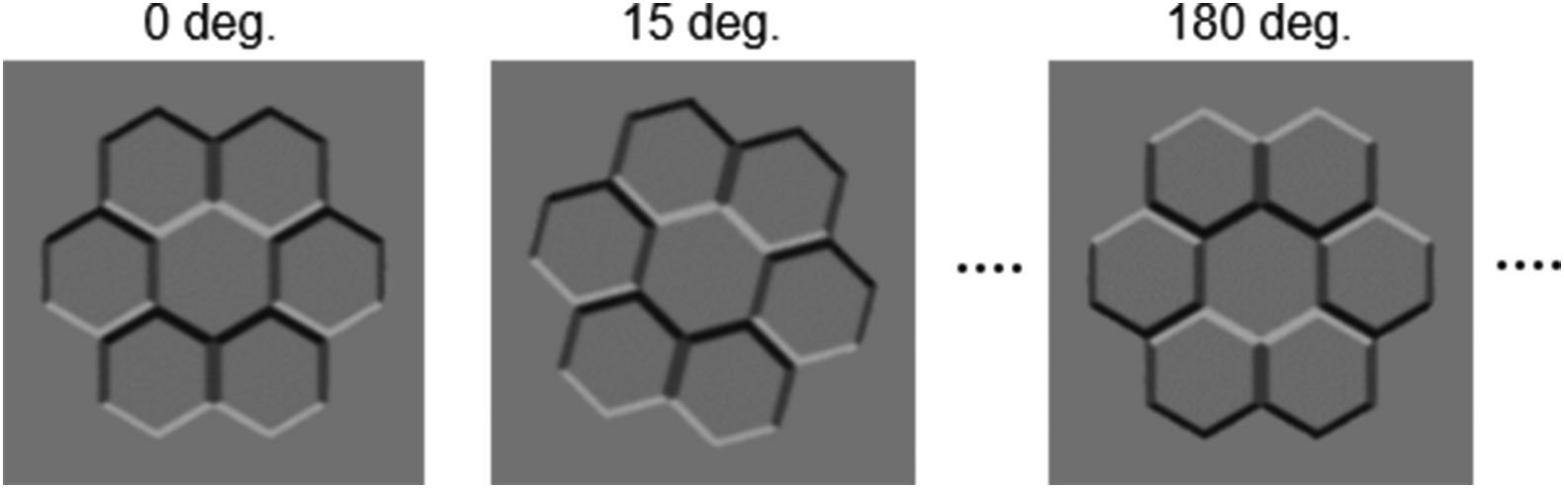
Examples of the “honeycomb” stimulus. Its orientation varied randomly in each trial.

Adapted from Andrews et al. (2013), this task used similar stimuli (Figure 2). This “honeycomb” configuration consists of seven hexagons with shaded edges to project a convex/concave impression. Assuming a single light source (Ramachandran, 1988), the central hexagon appears to be at an opposite depth to the surrounding six. This stimulus was considered to provide a more salient depth impression than the one generated by a conventional shaded hemisphere (Gerardin et al., 2007). Including the gray background, the stimulus image measured 13 cm in height and width on the screen (the height and width of the honeycomb image's area were approximately 10.3 and 10.6 cm, respectively).

The stimulus was presented at the center of the gray background. The background had the same shade of gray as the stimulus area (34.4 cd/m^2^). In each trial, the stimulus was presented at one of 24 equal-stepped angles from 0 degrees to 345 degrees.

The task involved pressing a key to determine whether the central hexagon appeared convex or concave. The presented stimulus was visible for 500 ms but was interrupted by the participants’ key press. After a trial was completed, only the background was shown for 500 ms before the next trial. Each angle condition was repeated 20 times. Therefore, this task consisted of 480 trials in random order. When the participants completed the 240th trial, they were allowed to take a break (and the participants could resume the task freely by pressing a key). Before the main task, the participants performed 32 practice trials under normal illumination without a feedback. Some participants who did not understand the task in this practice session performed it twice.

##### Lightness task

**Figure 3.**
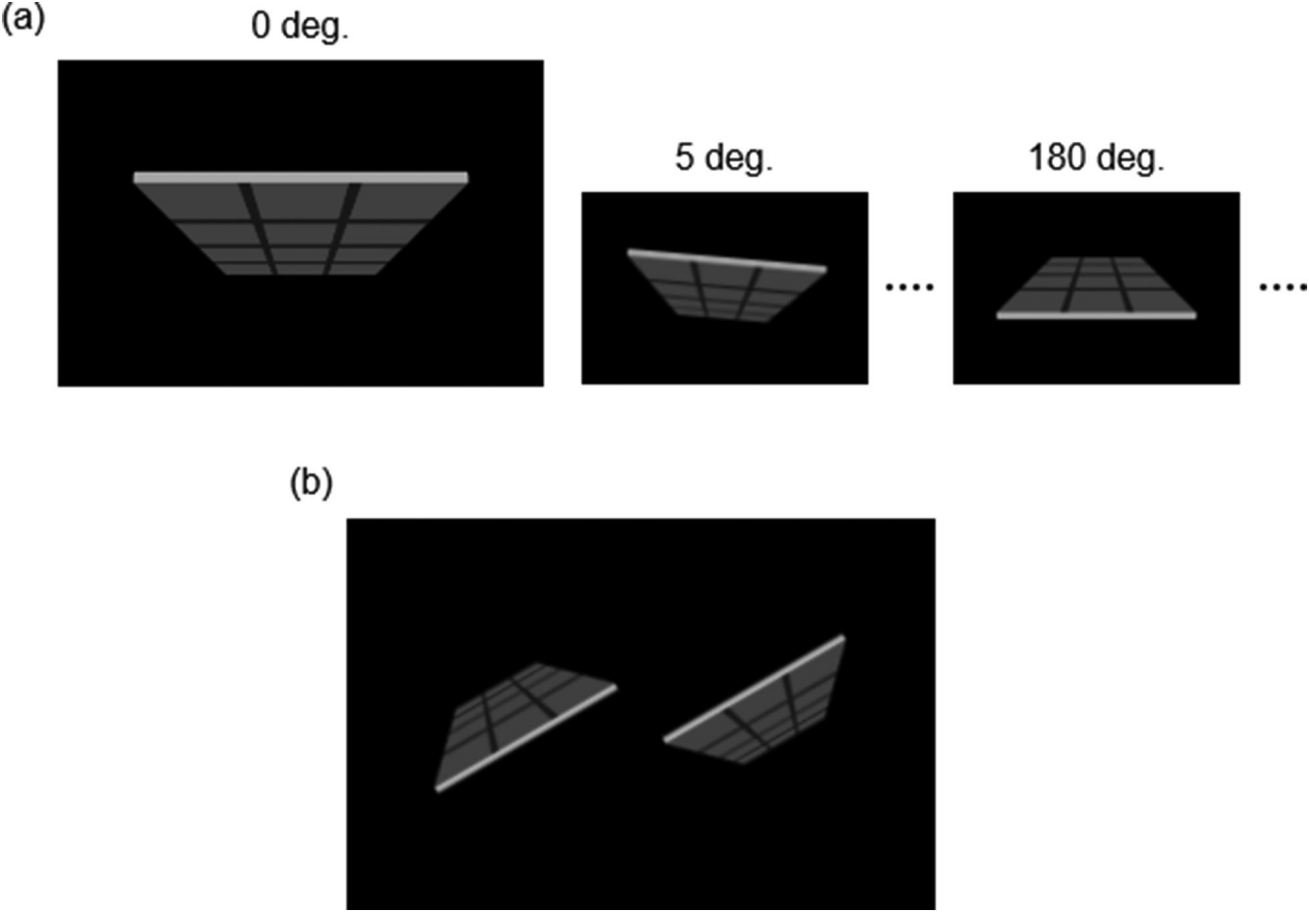
(**a**) Examples of the surface stimuli. (**b**) An example of the experiment display.

Because the objects in Figure 1 are asymmetric, they may introduce a systematic bias. To avoid this concern, a symmetric surface stimulus was devised (Figure 3a). It had grids on its gray surface to provide depth cues. With the black background, the stimulus was 9.6 cm high and 14.2 cm wide on the screen (the height and width of the panel image's area were approximately 3.1 and 9.9 cm, respectively), and its luminance was fixed (gray area = 12.2 cd/m^2^, black grids = 1.7 cd/m^2^, and white edge = 74.4 cd/m^2^).

A pair of stimuli was presented at the center of the black background. One image of each pair was a 180 degrees rotation of the other (Figure 3b). Thirty-six angle conditions at five-degree steps were prepared from 0 degrees to 175 degrees. The stimuli were presented at an angle randomly chosen from these conditions. The centers of the two stimuli were 11.9 cm apart.

By pressing a key, the participants chose which stimulus surface appeared darker. The stimuli were presented for 1500 ms but were interrupted by the key presses. After the presentation, a 500 ms blank was inserted before the next trial started.

The stimulus pair had no differences in physical luminance because they were the same image, but the upward-facing version was expected to be chosen more often because of the illusory effect of the light-from-above prior. Each angle condition was repeated 16 times; therefore, the whole lightness task session consisted of 576 trials. During the session, breaks were provided when the participants completed the 144th, 288th, and 432nd trials. Before the main task, the participants practiced with eight trials under normal illumination without a feedback. Some participants who wanted to redo the practice performed it twice.

### Results

Five participants were excluded from the analysis, four because their data did not show a good fit for a multivariate logistic regression as described in the following paragraphs (pseudo *R*^2^ < 0.10) and one because the trials were disrupted by a program crash. One participant reported during the instructions that he did not perceive convexity for any of the honeycomb stimuli and thus did not perform the main experiment. This left a total of 20 participants whose data were analyzed. One participant reported that he used opposite keys for concave and convex in the 3-D shape task; thus, his data were reversed in the analysis. In the 3-D shape task, a very small percentage of trials were interrupted by key presses before stimulus presentation. Therefore, these trials were excluded from the analysis (less than 0.01% of all trials).

**Figure 4.**
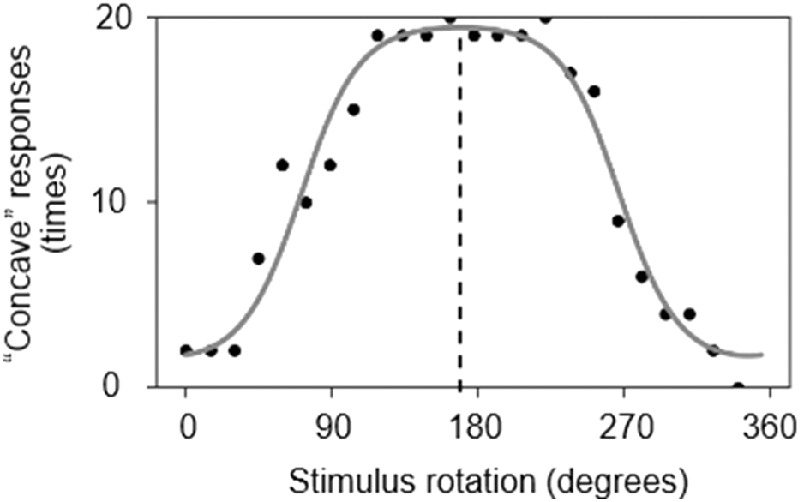
Data from one representative participant in the 3-D shape task. The black dots indicate the actual data, and the gray curve shows the model fit. When the honeycomb stimulus was upright (i.e., 0 degrees and 360 degrees), the target area was likely to be perceived as convex. If the participant's assumed lighting direction is directly above, the peak of the curve (e.g. the “most concave” rotation) should be at 180 degrees. The peak's shift from 180 degrees indicates a bias of assumed lighting direction. In these data, the peak is at approximately 172 degrees, indicating an 8 degrees bias toward the left.

Figure 4 illustrates a representative participant's data for the 3-D shape task, showing the number of “concave” responses as a function of angle condition. The black dots correspond to the actual data, and the gray curve is the calculated fit as explained below. The target area was likely to be perceived as convex at angles near 0 degrees and 360 degrees and as concave at around 180 degrees.

The logistic regression (gray curve) and the direction of assumed lighting were calculated based on the method used by Andrews et al. (2013). The curve was defined as follows:

(1) $$p\left(\mathrm{convex}|\theta \right)=\frac{1}{1+e^{-f\left(\theta \right)}}$$

where

(2) $$f\left(\theta \right)=a+b\cdot \cos\theta +c\cdot \sin\theta$$

Here, *θ* refers to the rotation angle of the honeycomb stimulus. The assumed lighting direction was defined this way:

(3) $$\mathrm{Direction}=\;\tan^{-1}\frac{c}{b}$$

If the direction is directly from above, the value is zero. A negative value corresponds to a bias toward the left.

**Figure 5.**
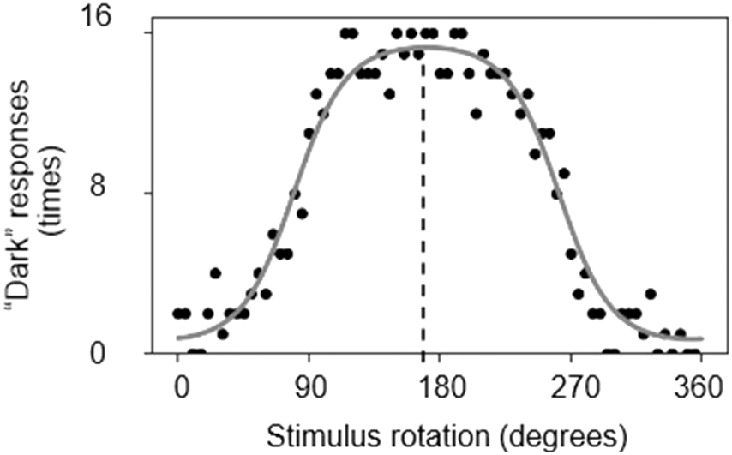
Data from one representative participant in the lightness task. The black dots indicate the actual data, and the gray curve shows the model fit. A downward-facing surface stimulus (0 degrees and 360 degrees) was likely to be perceived as lighter, and when it faced upward (180 degrees), it appeared darker. In this example, the peak is at approximately 168 degrees, which corresponds to a 12 degrees bias toward the left (i.e., the calculated direction value is −12).

Figure 5 shows the lightness task data from the same participant, indicating the selection rates (i.e., perceived as darker) at each rotation angle. Here, the selection rate for *x* degree (select [*x*]) is equal to {1 – select [*x* *+* 180]} (0 ≤ *x* < 180). Surfaces facing upward (near 180 degrees) were more likely to be chosen as “darker,” which replicated the result of Kobayashi and Morikawa (2019). For each participant's data, the fit and assumed direction were calculated using the same model in the 3-D shape task.

**Figure 6.**
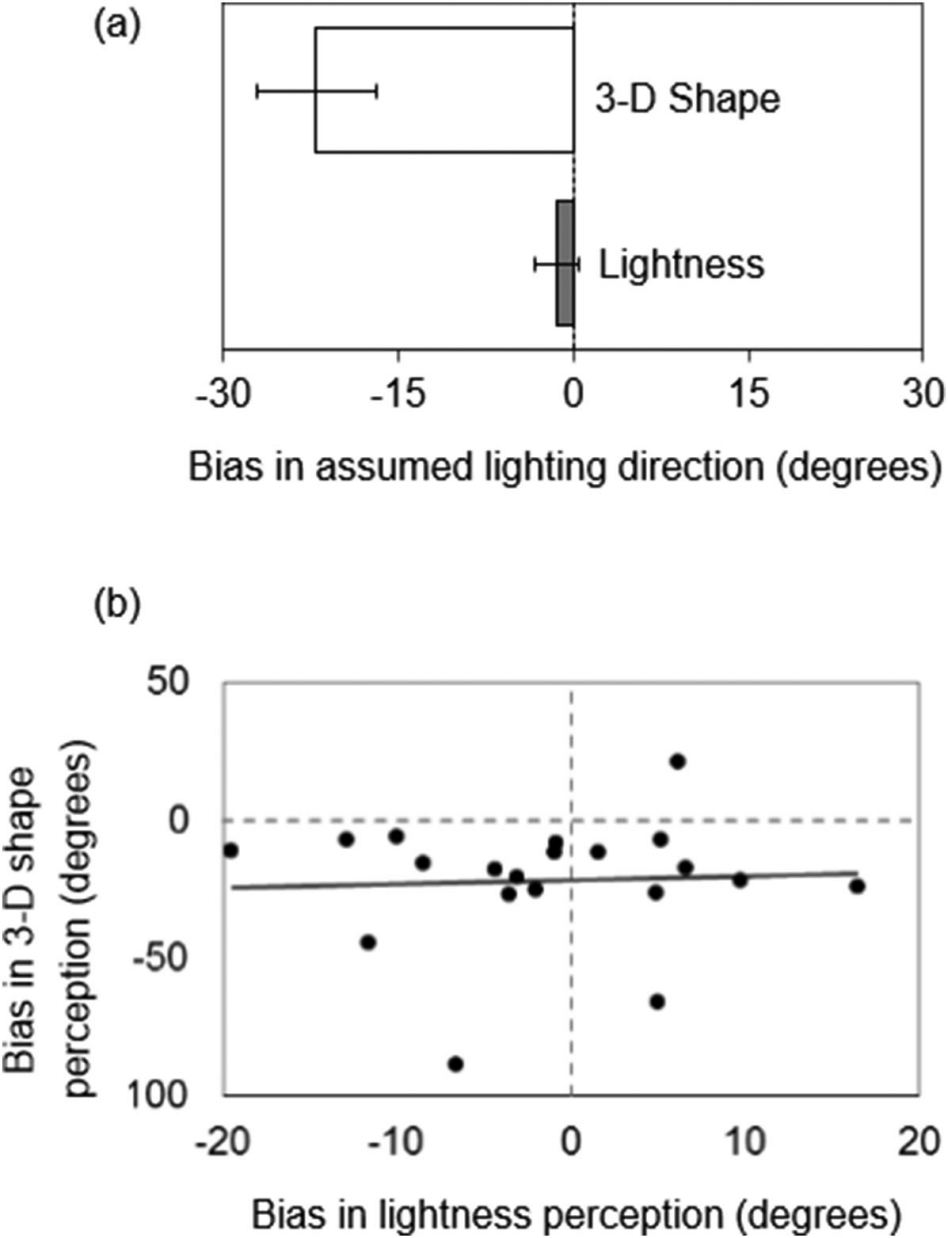
(**a**) Comparison of the averages of assumed lighting directions in Experiment 1. Zero means no bias, and negative values indicate a leftward bias. Error bars indicate SE. (**b**) Scatter plot of individual lighting directions in both tasks. The black line shows the linear fit.

Figure 6(a) shows the averages of assumed lighting directions extracted from the two tasks. The 3-D shape task showed a clear leftward bias for the assumed lighting direction (−22.0 degrees; *t*(19) = 4.27, *p* < 0.001, *d_z_* = 0.95),^1^ instead, results for the lightness task did not show such a bias (−1.4 degrees; *t*(19) = 0.73, *p* = 0.476, *d_z_* = 0.16). A pairwise *t*-test was also conducted to directly examine the angular difference between the lighting priors for the two tasks, although this test was not planned before the experiment. It resulted in a significant difference (*t*(19) = 3.80, *p* = 0.001, *d_av_* = 1.30). Along with the comparison of means, the correlation among participants was also examined. If 3-D shapes and lightness share lighting priors, the directions calculated based on the results of the two tasks should be positively correlated (i.e., a participant showing a strong leftward bias in 3-D shape perception would display the same in lightness perception). However, the data did not support this hypothesis (Figure 6b), and the correlation was not significant (*r* = 0.050, *p* = 0.836).

## Experiment 2

As previously mentioned, the writing/reading direction of the observers’ main language (e.g. Hebrew or English) was found to be a contributing factor to their assumed lighting direction (Andrews et al., 2013). Japanese is intermediate between Hebrew and English in that its text is read and written either from left to right (horizontal writing) or from up to down with lines progressing from right to left (vertical writing). In Japan, texts in novels, newspapers, comic books, and the majority of magazines and books for general readers are written vertically (and thus a page of a smaller number is on the right side of the spread in these publications). Therefore, although the consent forms and instructions in the present experiments were written horizontally, the experience of vertical reading/writing is very common among all Japanese participants. Reading/writing direction can affect different aspects of visual perception (Andrews et al., 2013; Ishii, Okubo, Nicholls, & Imai, 2011; Morikawa & McBeath, 1992). Although the Japanese vertical reading-from-right style is not similar to that of Hebrew's, the possibility of its influence should be examined. The lack of a leftward bias in the lightness task in Experiment 1 might be specific to Japanese people, who use both right-to-left vertical and left-to-right horizontal writing. Therefore, in Experiment 2, the same tasks were performed by European language speakers to examine the effects of the languages’ writing practices on the two lighting priors.

### Methods

#### Participants

Experiment 2 was conducted in the Department of Psychology at the University of Milano – Bicocca. It used 22 naïve participants from the said university who were 19 to 46 years old (*M* = 24.9, *SD* = 6.23, 18 women and 4 men). All their first languages were European (Italian, French, German, or English) except for one participant who was proficient in Tagalog, Italian, and English. All these languages use left-to-right writing and reading. One participant reported writing with their left hand. All participants had normal or corrected-to-normal visual acuity. As described in the Results section, two people were excluded from analysis because of inconsistent responses. Consequently, the final sample size (20) was the same as in Experiment 1.

#### Apparatus

A CRT monitor (LACIE Electron 22 blue II, Mitsubishi Electric Corporation) with a 1600 × 1200 resolution was used. The monitor screen was 41 cm wide and 31 cm high. A BM-7A (Topcon) was used to measure stimuli luminance. Viewing distance was fixed at 57 cm using a chin rest, and the experiment was conducted on PsychoPy3 (Peirce, 2007, 2009; Peirce et al., 2019). The participants performed the task in a dark room without any light sources except the monitor.

#### Stimuli and tasks

The stimuli and tasks were virtually the same as those in Experiment 1, with some minor differences. First, because of the different pixel size of the monitor used, the images were slightly larger: including the background, the honeycomb stimulus had a height and width of 13.3 cm (13 cm in Experiment 1). The surface stimulus image was 9.8 cm high and 14.8 cm wide (9.6 cm high and 14.2 cm wide in Experiment 1). The surface stimulus pair was 12.3 cm apart (11.9 cm in Experiment 1). Second, the stimuli had lower luminance values: the gray area in the 3-D shape task (the background and the large body of the honeycomb stimulus) was 7.3 cd/m^2^, and the surface stimuli were 2.3 cd/m^2^ on the gray surface, 0.3 cd/m^2^ on the black grids, and 16.7 cd/m^2^ on the white edge. This difference in luminance was due to the narrower luminance range of the monitor used in this experiment. Third, the practice session was conducted in the dark. Nevertheless, these differences were trivial and unlikely to affect the leftward or rightward bias of the light-from-above prior.

### Results

Two participants were excluded. For one, the data from the 3-D shape task could not be fitted with the logistic regression model (pseudo *R*^2^ < 0.10) whereas the other showed a strong concavity bias in the 3-D shape task (she chose “concave” in 97.3% of all the trials). Data from the remaining 20 participants were analyzed. A small percentage of trials were excluded from the 3-D shape task data because of interruptions before stimulus presentations (less than 0.01% of all trials).

**Figure 7.**
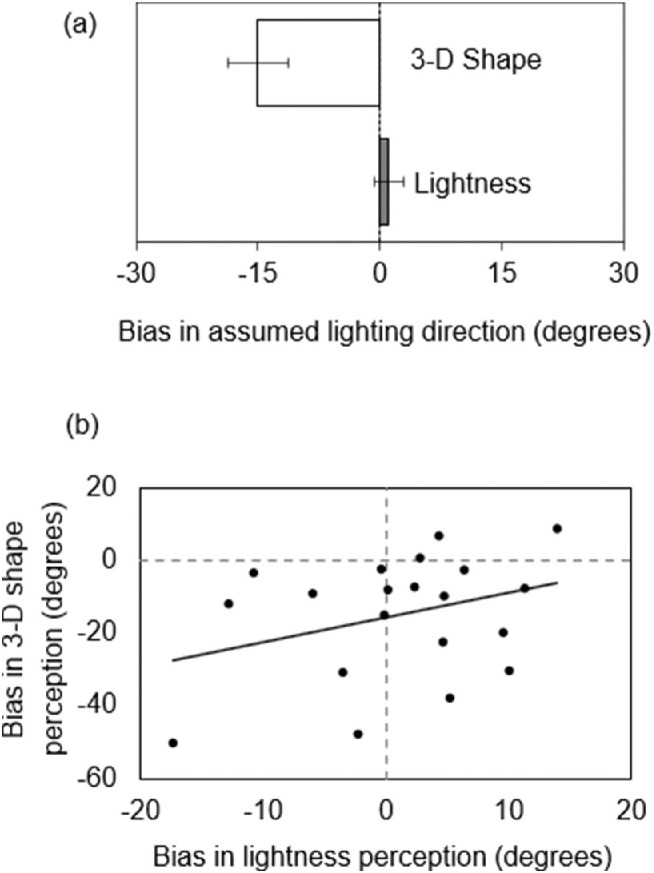
(**a**) Comparison of averages of assumed lighting biases in Experiment 2. Error bars indicate SE. (**b**) Scatter plot of individual lighting directions in both tasks. The black line shows the linear fit.

Figure 7 shows the results. The directions extracted from the 3-D shape and lightness tasks were −15.0 degrees and 1.1 degrees, respectively (Figure 7a), showing a significant difference (*t*(19) = 4.48, *p* < 0.001, *d_av_* = 1.29). Again, a leftward bias was robustly observed in the 3-D shape task (*t*(19) = 4.00, *p* < 0.001, *d_z_* = 0.89) but not in the lightness task (*t*(19) = 0.60, *p* = 0.555, *d_z_* = 0.13). The correlation among the participants was not significant (Figure 7b; *r* = 0.329, *p* = 0.156). These results replicated those of Experiment 1, confirming the independence of these two lighting priors. The data of the two experiments were combined and underwent a mixed-design analysis of variance (shape/lightness × languages), and neither the main effect of languages nor the interaction was found to be significant (shape/lightness: *F*(1, 38) = 31.9, *p* < 0.001, η_p_^2^ = 0.456; language: *F*(1, 38) = 1.70, *p* = 0.200, η_p_^2^ = 0.043; and interaction: *F*(1, 38) = 0.48, *p* = 0.493, η_p_^2^ = 0.012).

These analyses indicated that the results of the two experiments were essentially the same. Therefore, the lack of a leftward lighting bias for the lightness task in Experiment 1 is not attributed to Japanese speakers’ vertical writing experience. Moreover, in Experiment 2, the leftward bias of European language speakers was not stronger than that of Japanese speakers in both tasks, further reinforcing the irrelevance of reading/writing direction.

The results of Experiment 2 clearly confirmed those of Experiment 1. The difference between the two lighting priors was observed even among European language speakers. Taken together, these experiments support the robust independence of these two priors.

## Discussion

In Experiment 1, the two assumed lighting directions for 3-D shape and lightness perception from each participant were calculated and compared. The lighting prior used in 3-D shape perception showed a significant leftward bias, which is consistent with previous studies (e.g. Andrews et al., 2013; Sun & Perona, 1998). In lightness perception, however, the lighting prior was almost directly overhead with no leftward bias. Moreover, the two assumed lighting directions did not correlate with each other; that is, the individual differences in one prior did not explain those in the other. These results were then replicated by Experiment 2, whose participants only used languages written/read from left to right (European and Tagalog). All the results of the present experiments support the hypothesis that the priors used for 3-D shape and lightness perception are independent of each other.

Studies have also suggested a difference in illumination processing between 3-D shape and lightness perception. Morgenstern, Murray, and Harris (2011) showed that the addition of illumination cues in the scene can alter convex/concave perception. This finding suggests that the lighting prior used for 3-D shape perception can be overridden by visual illumination information. As for the lightness perception, the effect of illumination has been debatable. For example, Boyaci's group (Boyaci, Doerschner, Snyder, & Maloney, 2006; Boyaci, Maloney, & Hersh, 2003) showed that the lightness judgment varies in accordance with the target surface's orientation to the light source, whose position and direction are indicated by highlights and shadows of surrounding objects. However, Gilchrist (2018) argued that Boyaci et al. (2003) results can be explained by the relaxed coplanar principle (Gilchrist, 1977), which hypothesizes that lightness is determined by luminance ratios with surrounding surfaces, and this effect is stronger as the surrounding surface becomes more parallel to the target plane. This observation suggests that the estimates of illumination need not be considered explicitly to explain Boyaci et al. results. In lightness studies, there have been discussions regarding the role of illumination estimates (e.g. Pont & Koenderink, 2007; Todorović, 2006; Zavagno, Daneyko, & Liu, 2018); however, researchers’ opinions are inconsistent. Therefore, whereas studies show that the 3-D shape perception is certainly susceptible to visual illumination cues, the lightness perception may not. This suggests the different roles of illumination information in these two visual processes.

We can only ecologically speculate why the visual system holds two distinct lighting priors. Perhaps the lighting prior for the 3-D shape perception needs to be updated more rapidly than the one for the lightness perception. Suppose you are running as fast as you can over an uneven terrain either chasing prey or running away from a predator. Misperceiving a bump on the ground for a depression could result in fatal stumbling, and the shading of the bump/depression varies from hour to hour. To accurately perceive a shape, the visual system must frequently update the lighting prior. That may be why the lighting prior for the 3-D shape perception is biased toward the left of vertical, which may be caused by most people's use of the right hand for writing and manipulation (Sun & Perona, 1998). By contrast, changes in the perceived lightness caused by the sun's direction are more subtle, more gradual, and seldom fatal. Therefore, for the lightness perception, the visual system may fall back on the default lighting prior (i.e., directly overhead).

Adams et al. (2004) work is highly relevant to the current study. Their Experiment 2 used the “cocktail-stick” stimuli, and the results showed that the upward-facing side of a convex object appeared darker in pigment than the downward-facing side of the same luminance. These findings are similar to the lightness inversion effect demonstrated by Kobayashi and Morikawa (2019), except that the former was contingent upon the convexity of the stimulus and the latter was not. In particular, Adams et al. (2004) reported that cross-modal adaptation with haptic stimuli shifted the orientation of lighting priors for both convex/concave shape judgments and lightness judgments. At the first glance, their results may imply a common lighting prior for shape and lightness, thereby contradicting our results. However, their findings are not necessarily inconsistent with the present study because separate lighting priors for the shape and lightness perceptions may be independently susceptible to haptic influences. This speculation is supported because a haptic feedback is sufficiently powerful to change the visual perception when visual stimuli are ambiguous (Ernst, Banks, & Bülthoff, 2000; Wijntjes, Volcic, & Pont, 2009). Furthermore, because Adams et al. (2004) used convex objects for lightness judgments, their lightness perception task may have included a factor of the convex/concave shape perception unlike the present experiments. Therefore, Adams et al. (2004) results are highly relevant to but not necessarily inconsistent with the current findings.

Unlike the present study, Adams (2007) found a positive correlation between the assumed lighting directions from the 3-D shape perception task and a reflectance judgment task. The discrepancy with our results seems to stem from the different characteristics of our lightness perception task and her reflectance judgment task. Although both tasks focused on surface tone, their requirements were substantially different; whereas we simply asked participants to choose a darker surface, Adams's task required determining which of two three-sided objects appeared to be uniformly colored with the same pigment. The objects in Adams's experiment were explicitly illuminated, and illumination interpretation was necessary to perform the task, thus producing results different from those of the present study. Our results clarified the lighting prior used in lightness perception in the absence of illumination cues.

Moreover, Adams (2007) reflectance-judgment task was contingent upon the perception of the convexity of the tetrahedrons. Although Adams added a piercing ring to the stimuli to strengthen their convex appearance, its effectiveness was not guaranteed. For example, when the tetrahedron was lit from below (i.e., the bottom surface is brighter), the object could be considered concave despite the ring, as in her Figure 1(c) right. Therefore, her task included a factor of shape from shading similar to the other two tasks (i.e., visual search and shape perception). Moreover, the explicit illumination cues used in her study may have imposed some constraints on the range of the estimated direction of the light source. Therefore, all the three tasks may have involved common processing, and the reflectance-judgment task may have been subjected to the same constraints as the lighting prior in visual search and shape perception. Then, as anticipated, Adams (2007) found positive correlations among the three types of measurement. By contrast, the present study's reflectance-judgment task did not involve convexity/concavity issues or illumination cues. Therefore, the assumed light direction was probably measured in a constraint-free manner. This may be why the present study's results are different from those of Adams.

The inversion effect in lightness (see Figure 1) is relatively weak. Figure 1 shows a smaller difference in perceived lightness than conventional lightness illusions such as simultaneous lightness contrast or White's effect (White, 1979). Such weakness might suggest the diffuseness of assumed lighting for lightness perception because diffuse lighting would cause a smaller difference in illuminance between upward-facing and downward-facing surfaces than a point light source. Although previous studies have shown that natural lighting is often diffuse *and* directional (Morgenstern, Geisler, & Murray, 2014), we do not yet know how diffuse the lighting priors for the 3-D shape and lightness perceptions are. The diffuseness of assumed lighting has not attracted as much attention as its direction; however, it is also worth investigating (Langer & Bülthoff, 2000; Morgenstern, Geisler, & Murray, 2015; Murray & Adams, 2019; Stone, Kerrigan, & Porrill, 2009; Xia, Pont, & Heynderickx, 2017a, 2017b, 2017c).

The current findings have theoretical implications for the levels of lightness and 3-D shape processing. Numerous studies have shown that the lighting prior for the 3-D shape perception is modified by experience (Adams et al., 2010; Kerrigan & Adams, 2013; Stone, 2011; Stone & Pascalis, 2010; Thomas et al., 2010), which suggests that the 3-D shape perception is a relatively high-level process. On the other hand, our results show that the lighting prior for the lightness perception is independent of that for the 3-D shape perception. However, very few studies have examined whether the lighting prior for the lightness perception is modified by experience (Adams et al., 2004). If it is not, the lightness perception may be a mid-level process, which is lower than the 3-D shape perception (Gilchrist, 2006; Kobayashi & Morikawa, 2019). Further research would clarify the relative levels of lightness and 3-D shape processing.

Because the inversion effect in lightness is a relatively new phenomenon, not much is known about its mechanism. It is possible that some low-level features in images may also contribute to the inversion effect of lightness (Kobayashi & Morikawa, 2020), which would corroborate the present findings in that the process underlying the inversion effect is not the same as that of the shape-from-shading phenomenon. Future research must further investigate and explain the mechanism that causes this vertical asymmetry of lightness perception.

## Conclusions

This study investigated whether the perceptual processes of the 3-D shape and lightness share a common lighting prior or use independent priors. The two experiments revealed a substantial and robust difference between the two assumed lighting directions obtained from the 3-D shape and lightness perception tasks, thereby supporting the independent-priors hypothesis.

## Supplement

The data, stimuli, and Python codes used for analyses are available at: https://doi.org/10.17605/OSF.IO/AKNJ2.

## Footnotes

1 In this paper, *d_z_* and *d_av_* are reported as the effect sizes of *t* tests based on Lakens (2013).

## References

[bib1] Adams, W. J., Graf, E. W., & Ernst, M. O. (2004). Experience can change the 'light-from-above' prior. *Nature Neuroscience,* 7, 1057–1058. 15361877 10.1038/nn1312

[bib2] Adams, W. J. (2007). A common light-prior for visual search, shape, and reflectance judgments. *Journal of Vision,* 7(11), 1–7. 10.1167/7.11.11 17997666

[bib3] Adams, W. J. (2008). Frames of reference for the light-from-above prior in visual search and shape judgements. *Cognition,* 107(1), 137–150. 17950264 10.1016/j.cognition.2007.08.006

[bib4] Adams, W. J., Kerrigan, I. S., & Graf, E. W. (2010). Efficient visual recalibration from either visual or haptic feedback: the importance of being wrong. *Journal of Neuroscience,* 30(44), 14745–14749. 21048133 10.1523/JNEUROSCI.2749-10.2010 PMC6633618

[bib6] Andrews, B., Aisenberg, D., d'Avossa, G., & Sapir, A. (2013). Cross-cultural effects on the assumed light source direction: Evidence from English and Hebrew readers. *Journal of Vision,* 13(13), 1–7. 10.1167/13.13.2 24187057

[bib7] Anderson, B. L., & Kim, J. (2009). Image statistics do not explain the perception of gloss and lightness. *Journal of Vision,* 9(11), 1–17. 10.1167/9.11.10 20053073

[bib8] Boyaci, H., Doerschner, K., Snyder, J. L., & Maloney, L. T. (2006). Surface color perception in three-dimensional scenes. *Visual Neuroscience,* 23, 311–321. 16961962 10.1017/S0952523806233431

[bib9] Boyaci, H., Maloney, L. T., & Hersh, S. (2003). The effect of perceived surface orientation on perceived surface albedo in binocularly viewed scenes. *Journal of Vision,* 3(8), 541–553. 14632606 10.1167/3.8.2

[bib10] Ernst, M. O., Banks, M. S., & Bülthoff, H. H. (2000). Touch can change visual slant perception. *Nature Neuroscience,* 3(1), 69–73. 10607397 10.1038/71140

[bib11] Gerardin, P., de Montalembert, M., & Mamassian, P. (2007). Shape from shading: New perspectives from the Polo Mint stimulus. *Journal of Vision,* 7(11), 1–11. 10.1167/7.11.13 18050885

[bib11a] Gilchrist, A. L. (1977). Perceived lightness depends on perceived spatial arrangement. *Science,* 195(4274), 185–187. 831266 10.1126/science.831266

[bib12] Gilchrist, A. L. (2006). *Seeing black and white*. Oxford, UK: Oxford University Press.

[bib13] Gilchrist, A. L. (2018). To compute lightness, illumination is not estimated, it is held constant. *Journal of Experimental Psychology: Human Perception and Performance,* 44(8), 1258–1267. 29723008 10.1037/xhp0000487 PMC6062464

[bib14] Ishii, Y., Okubo, M., Nicholls, M. E., & Imai, H. (2011). Lateral biases and reading direction: A dissociation between aesthetic preference and line bisection. *Brain and Cognition,* 75(3), 242–247. 21215506 10.1016/j.bandc.2010.12.005

[bib15] Kerrigan, I. S., & Adams, W. J. (2013). Learning different light prior distributions for different contexts. *Cognition,* 127(1), 99–104. 23376295 10.1016/j.cognition.2012.12.011

[bib16] Knill, D. C., & Kersten, D. (1991). Apparent surface curvature affects lightness perception. *Nature,* 351(6323), 228. 2041568 10.1038/351228a0

[bib17] Kobayashi, Y., & Morikawa, K. (2019). An upward-facing surface appears darker: the role played by the light-from-above assumption in lightness perception. *Perception,* 48(6), 500–514. 31084253 10.1177/0301006619847590

[bib18] Kobayashi, Y., & Morikawa, K. (2020, November 19-22). *Vertical Anisotropy in Lightness Perception: An Inversion Effect of Lightness Not* *Caused by Lighting Prior*. [Conference presentation abstract]. 61st Annual Meeting of Psychonomic Society, Online. https://cdn.ymaws.com/www.psychonomic.org/resource/resmgr/annual_meeting/2020_meeting/2020_abstract_book/PS20_Abstracts_V10_linked_lo.pdf.

[bib19] Lakens, D. (2013). Calculating and reporting effect sizes to facilitate cumulative science: a practical primer for t-tests and ANOVAs. *Frontiers in Psychology,* 4, 1–12. 24324449 10.3389/fpsyg.2013.00863 PMC3840331

[bib20] Langer, M. S., & Bülthoff, H. H. (2000). Depth discrimination from shading under diffuse lighting. *Perception,* 29(6), 649–660. 11040949 10.1068/p3060

[bib21] Mamassian, P., & Goutcher, R. (2001). Prior knowledge on the illumination position. *Cognition,* 81(1), B1–B9. 11525484 10.1016/s0010-0277(01)00116-0

[bib22] Marlow, P. J., Kim, J., & Anderson, B. L. (2017). Perception and misperception of surface opacity. *Proceedings of the National Academy of Sciences,* 114(52), 13840–13845. 10.1073/pnas.1711416115 PMC5748181 29229812

[bib23] Marlow, P. J., Mooney, S. W., & Anderson, B. L. (2019). Photogeometric cues to perceived surface shading. *Current Biology,* 29(2), 306–311. 30612905 10.1016/j.cub.2018.11.041

[bib24] Marlow, P. J., Todorović, D., & Anderson, B. L. (2015). Coupled computations of three-dimensional shape and material. *Current Biology,* 25(6), R221–R222. 25784037 10.1016/j.cub.2015.01.062

[bib25] McManus, C., Buckman, J., & Wooley, E. (2004). Is light in pictures presumed to come from the left side? *Perception,* 33(12), 1421–1436. 15729910 10.1068/p5289

[bib26] Menshikova, G. Y. (2013). An investigation of 3D images of the simultaneous-lightness-contrast illusion using a virtual-reality technique. *Psychology in Russia: State of the Art,* 6(3), 49–59.

[bib27] Metzger, W. (2006). *Laws of seeing* (L. Spillmann, Trans.). Cambridge, MA: MIT Press. (Original work published 1936).

[bib28] Morgenstern, Y., Geisler, W. S., & Murray, R. F. (2014). Human vision is attuned to the diffuseness of natural light. *Journal of Vision,* 14(9), 15. 10.1167/14.9.15 PMC4141658 25139864

[bib29] Morgenstern, Y., Geisler, W. S., & Murray, R. F. (2015). The role of natural lighting diffuseness in human visual perception. In *Human Vision and Electronic Imaging XX* (Vol. 9394, p. 93940P). Bellingham, WA: International Society for Optics and Photonics.

[bib30] Morgenstern, Y., Murray, R. F., & Harris, L. R. (2011). The human visual system's assumption that light comes from above is weak. *Proceedings of the National Academy of Sciences,* 108(30), 12551–12553. 10.1073/pnas.1100794108 PMC3145687 21746935

[bib31] Morikawa, K., & McBeath, M. K. (1992). Lateral motion bias associated with reading direction. *Vision Research,* 32(6), 1137–1141. 1509704 10.1016/0042-6989(92)90014-a

[bib32] Murray, R. F., & Adams, W. J. (2019). Visual perception and natural illumination. *Current Opinion in Behavioral Sciences**,* 30, 48–54.

[bib33] Peirce, J. W. (2007). PsychoPy—psychophysics software in Python. *Journal of Neuroscience Methods,* 162(1-2), 8–13. 17254636 10.1016/j.jneumeth.2006.11.017 PMC2018741

[bib34] Peirce, J. W. (2009). Generating stimuli for neuroscience using PsychoPy. *Frontiers in Neuroinformatics,* 2, 1–8. 10.3389/neuro.11.010.2008 PMC2636899 19198666

[bib35] Peirce, J. W., Gray, J. R., Simpson, S., MacAskill, M. R., Höchenberger, R., Sogo, H., Kastman, E., & Lindeløv, J. (2019). PsychoPy2: experiments in behavior made easy. *Behavior Research Methods,* 51(1)*,* 195–203. 30734206 10.3758/s13428-018-01193-y PMC6420413

[bib36] Pont, S. C., & Koenderink, J. J. (2007). Matching illumination of solid objects. *Perception & Psychophysics,* 69(3), 459–468. 17672433 10.3758/bf03193766

[bib37] Ramachandran, V. S. (1988). Perception of shape from shading. *Nature,* 331(6152), 163–166. 3340162 10.1038/331163a0

[bib38] Stone, J. V. (2011). Footprints sticking out of the sand. Part 2: Children's Bayesian priors for shape and lighting direction. *Perception,* 40(2), 175–190. 21650091 10.1068/p6776

[bib39] Stone, J. V., Kerrigan, I. S., & Porrill, J. (2009). Where is the light? Bayesian perceptual priors for lighting direction. *Proceedings of the Royal Society B: Biological Sciences,* 276(1663), 1797–1804. 10.1098/rspb.2008.1635 PMC2674484 19324801

[bib40] Stone, J. V., & Pascalis, O. (2010). Footprints sticking out of the sand. Part 1: Children's perception of naturalistic and embossed symbol stimuli. *Perception,* 39(9), 1254–1260. 21125952 10.1068/p6725

[bib41] Sun, J., & Perona, P. (1998). Where is the sun? *Nature Neuroscience,* 1(3), 183–184. 10195141 10.1038/630

[bib42] Thomas, R., Nardini, M., & Mareschal, D. (2010). Interactions between “light-from-above” and convexity priors in visual development. *Journal of Vision,* 10(8), 1–7. 10.1167/10.8.6 20884581

[bib43] Todorović, D. (2006). Lightness, illumination, and gradients. *Spatial Vision,* 19(2–4), 219–261. 16862841 10.1163/156856806776923407

[bib44] van Doorn, A. J., Koenderink, J. J., Todd, J. T., & Wagemans, J. (2012). Awareness of the Light Field: The Case of Deformation. *i-Perception,* 3(7), 467–480. 23145298 10.1068/i0504 PMC3485835

[bib45] White, M. (1979). A new effect of pattern on perceived lightness. *Perception,* 8(4), 413–416. 503772 10.1068/p080413

[bib46] Wijntjes, M. W. A., Volcic, R., Pont, S. C., Koenderink, J. J., & Kappers, A. M. L. (2009). Haptic perception disambiguates visual perception of 3D shape. *Experimental Brain Research,* 193(4), 639–644. 19199097 10.1007/s00221-009-1713-9

[bib47] Witzel, C., & Hansen, T. (2015). Memory effects on color perception. A. J. Elliot, M. D. Fairchild, & A. Franklin (Eds.), *Handbook of Color Psychology* (pp. 641–659). Cambridge, MA: Cambridge University Press.

[bib48] Xia, L, Pont, S. C., & Heynderickx, I. (2017a). Light diffuseness metric Part 1: Theory. *Lighting Research & Technology,* 49(4), 411–427.

[bib49] Xia, L, Pont, S.C., & Heynderickx, I. (2017b). Light diffuseness metric, Part 2: Describing, measuring and visualising the light flow and diffuseness in three-dimensional spaces. *Lighting Research & Technology,* 49(4), 428–445.

[bib50] Xia, L, Pont, S. C., & Heynderickx, I. (2017c). Separate and simultaneous adjustment of light qualities in a real scene. *i-Perception,* 8(1). 10.1177/2041669516686089 PMC5298488 28203350

[bib51] Zavagno, D., Daneyko, O., & Liu, Z. (2018). The influence of physical illumination on lightness perception in simultaneous contrast displays. *i-Perception,* 9(4). 10.1177/2041669518787212 PMC6055112 30046432

